# Postoperative pain management in non-traumatic emergency general surgery: WSES-GAIS-SIAARTI-AAST guidelines

**DOI:** 10.1186/s13017-022-00455-7

**Published:** 2022-09-21

**Authors:** Federico Coccolini, Francesco Corradi, Massimo Sartelli, Raul Coimbra, Igor A. Kryvoruchko, Ari Leppaniemi, Krstina Doklestic, Elena Bignami, Giandomenico Biancofiore, Miklosh Bala, Ceresoli Marco, Dimitris Damaskos, Walt L. Biffl, Paola Fugazzola, Domenico Santonastaso, Vanni Agnoletti, Catia Sbarbaro, Mirco Nacoti, Timothy C. Hardcastle, Diego Mariani, Belinda De Simone, Matti Tolonen, Chad Ball, Mauro Podda, Isidoro Di Carlo, Salomone Di Saverio, Pradeep Navsaria, Luigi Bonavina, Fikri Abu-Zidan, Kjetil Soreide, Gustavo P. Fraga, Vanessa Henriques Carvalho, Sergio Faria Batista, Andreas Hecker, Alessandro Cucchetti, Giorgio Ercolani, Dario Tartaglia, Joseph M. Galante, Imtiaz Wani, Hayato Kurihara, Edward Tan, Andrey Litvin, Rita Maria Melotti, Gabriele Sganga, Tamara Zoro, Alessandro Isirdi, Nicola De’Angelis, Dieter G. Weber, Adrien M. Hodonou, Richard tenBroek, Dario Parini, Jim Khan, Giovanni Sbrana, Carlo Coniglio, Antonino Giarratano, Angelo Gratarola, Claudia Zaghi, Oreste Romeo, Michael Kelly, Francesco Forfori, Massimo Chiarugi, Ernest E. Moore, Fausto Catena, Manu L. N. G. Malbrain

**Affiliations:** 1 General, Emergency and Trauma Surgery Department, Pisa University Hospital, Via Paradisa, 2, 56124 Pisa, Italy; 2 ICU Department, Pisa University Hospital, Pisa, Italy; 3 General Surgery Department, Macerata Hospital, Macerata, Italy; 4 Trauma Surgery Department, Riverside University Health System Medical Center, Loma Linda, CA USA; 5 Department of Surgery No2, Kharkiv National Medical University, Kharkiv, Ukraine; 6 General Surgery Department, Helsinki University Hospital, Helsinki, Finland; 7 Clinic of Emergency Surgery, University Clinical Center of Serbia, Belgrade, Serbia; 8 ICU Department, Parma University Hospital, Parma, Italy; 9 Transplant ICU Department, Pisa University Hospital, Pisa, Italy; 10 Trauma and Acute Care Surgery Unit Hadassah, Hebrew University Medical Center, Jerusalem, Israel; 11 General Surgery Department, Monza University Hospital, Monza, Italy; 12 General and Emergency Surgery, Royal Infirmary of Edinburgh, Edinburgh, UK; 13 Trauma/Acute Care Surgery, Scripps Clinic Medical Group, La Jolla, CA USA; 14 General Surgery Department, Pavia University Hospital, Pavia, Italy; 15 ICU Department, Bufalini Hospital, Cesena, Italy; 16 ICU Department Papa Giovanni XXIII Hospital, Bergamo, Italy; 17 Trauma and Burn Service, Inkosi Albert Luthuli Central Hospital, Mayville, Durban, South Africa; 18 General Surgery Department, Legnano Hospital, Legnano, Milano, Italy; 19 Emergency and Colorectal Surgery, Poissy and Saint Germain en Laye Hospitals, Poissy, France; 20 Emergency Surgery, HUS Helsinki University Hospital, Meilahti Tower Hospital, Helsinki, Finland; 21 Trauma and Acute Care Surgery, Foothills Medical Center, Calgary, AB Canada; 22 Department of Surgical Science, University of Cagliari, Cagliari, Italy; 23 General Surgery, Cannizzaro University Hospital, Catania, Italy; 24 General Surgery Department, San Benedetto del Tronto Hospital, San Benedetto del Tronto, Italy; 25 Trauma Center, Groote Schuur Hospital, University of Cape Town, Cape Town, South Africa; 26 General Surgery Department, San Donato Hospital, Milan, Italy; 27 Department of Surgery, College of Medicine and Health Sciences, United Arab Emirates University, Al-Ain, United Arab Emirates; 28 Department of Gastrointestinal Surgery, Stavanger University Hospital, University of Bergen, Bergen, Norway; 29 Division of Trauma Surgery, School of Medical Sciences, University of Campinas, Campinas, Brazil; 30 ICU Department, School of Medical Sciences, University of Campinas, Campinas, Brazil; 31 Anesthesia Department, School of Medical Sciences, Campinas, Brazil; 32 General Surgery, Giessen University Hospital, Giessen, Germany; 33 Department of Medical and Surgical Sciences – DIMEC, Alma Mater Studiorum - University of Bologna, General Surgery of the Morgagni - Pierantoni Hospital, Forlì, Italy; 34 General Surgery Department, UCLA Davis University Hospital, Los Angeles, CA USA; 35 General Surgery Department, Government Gousiua Hospital, Srinagar, India; 36 Emergency and Trauma Surgery Department, Milano University Hospital, Milan, Italy; 37 Emergency Department, Nijmegen Hospital, Nijmegen, The Netherlands; 38 Department of Surgical Disciplines, Immanuel Kant Baltic Federal University, Regional Clinical Hospital, Kaliningrad, Russia; 39 Bologna University, Bologna, Italy; 40 Fondazione Policlinico Universitario A. Gemelli IRCCS, Università Cattolica del Sacro Cuore, Rome, Italy; 41 Service de Chirurgie Digestive Et Hépato-Bilio-Pancréatique, Hôpital Henri Mondor, Université Paris Est, Créteil, France; 42 Department of General Surgery, Royal Perth Hospital, Perth, Australia; 43 Faculty of Medicine of Parakou, University of Parakou, Parakou, Benin; 44 General Surgery Department, Nijmegen Hospital, Nijmegen, The Netherlands; 45 General Surgery Department, Santa Maria Della Misericordia Hospital, Rovigo, Italy; 46 University of Portsmouth, Portsmouth Hospitals University NHS Trust UK, Portsmouth, UK; 47 ICU-HEMS Department, Arezzo Hospital, Arezzo, Italy; 48 ICU Department, Maggiore Hospital, Bologna, Italy; 49 ICU Department, P. Giaccone Hospital, Palermo, Italy; 50 ICU Department, San Martino Hospital, Genoa, Italy; 51 General, Emergency and Trauma Surgery Department, Vicenza Hospital, Vicenza, Italy; 52 Trauma and Surgical Critical Care, East Medical Center Drive, University of Michigan Health System, Ann Arbor, MI USA; 53 Department of General Surgery, Albury Hospital, Albury, Australia; 54 E. Moore Shock and Trauma Centre, Denver, CO USA; 55 General, Emergency and Trauma Surgery Department, Bufalini Hospital, Cesena, Italy; 56 First Department Anaesthesiology Intensive Therapy, Medical University Lublin, Lublin, Poland; 57 International Fluid Academy, Lovenjoel, Belgium

**Keywords:** Morbidity, Acute, Pain, Treatment, Emergency, Surgery, Acute

## Abstract

**Background:**

Non-traumatic emergency general surgery involves a heterogeneous population that may present with several underlying diseases. Timeous emergency surgical treatment should be supplemented with high-quality perioperative care, ideally performed by multidisciplinary teams trained to identify and handle complex postoperative courses. Uncontrolled or poorly controlled acute postoperative pain may result in significant complications. While pain management after elective surgery has been standardized in perioperative pathways, the traditional perioperative treatment of patients undergoing emergency surgery is often a haphazard practice. The present recommended pain management guidelines are for pain management after non-traumatic emergency surgical intervention. It is meant to provide clinicians a list of indications to prescribe the optimal analgesics even in the absence of a multidisciplinary pain team.

**Material and methods:**

An international expert panel discussed the different issues in subsequent rounds. Four international recognized scientific societies: World Society of Emergency Surgery (WSES), Global Alliance for Infection in Surgery (GAIS), Italian Society of Anesthesia, Analgesia Intensive Care (SIAARTI), and American Association for the Surgery of Trauma (AAST), endorsed the project and approved the final manuscript.

**Conclusion:**

Dealing with acute postoperative pain in the emergency abdominal surgery setting is complex, requires special attention, and should be multidisciplinary. Several tools are available, and their combination is mandatory whenever is possible. Analgesic approach to the various situations and conditions should be patient based and tailored according to procedure, pathology, age, response, and available expertise. A better understanding of the patho-mechanisms of postoperative pain for short- and long-term outcomes is necessary to improve prophylactic and treatment strategies.

## Introduction

Non-traumatic emergency general surgery involves a heterogeneous disease spectrum and a varied that may present with several underlying diseases [1]. In the last decades, this cohort of patients has progressively included more vulnerable, frail, and elderly people. Hypovolemia, hypoxia, sepsis, and most often severe pain are frequent, and the perioperative treatment may be challenging [2]. The surgical treatment should be supplemented by high-quality perioperative care, ideally performed by multidisciplinary teams trained to identify and handle complex postoperative courses [3]. Acute postoperative pain (APP) is still a major burden for most healthcare systems. About 70% of the 240 million postsurgical patients every year suffer from moderate-to-severe pain [4]. Uncontrolled APP may result in significant clinical and psychological changes that may be associated with higher subsequent risk of several medical complications due to immobility, poor respiratory mobility, and failure of nutritional progress, including pneumonia, infections, deep vein thrombosis, cardiovascular events, and depression [5]. Pain relief is fundamental in multimodal strategies to improve surgical outcome, together with preoperative assessment, information and optimization, reduction of surgical stress, rapid mobilization, and early oral nutrition [6]. The APP management may be pursued by several professionals and in many places a multidisciplinary team is not available. For this reason, precise indications must be provided to physicians managing postsurgical patients and specifically to emergency general surgeons to implement their tools in assisting emergency surgical patients.

International and national guidelines recommend in case of moderate-to-severe pain a few analgesic techniques, including intravenous (i.v.), per oral (p.o.) or subcutaneous (s.c.) routes, as well as epidural analgesia (EA), patient-controlled analgesia (PCA), and continuous peripheral nerve blocks (CPNB) [7]. Administration may aim for both local pain control and systemic effects, often with a combination to achieve optimal results. While an appropriate pain management has been optimized in the perioperative pathways after elective surgery, the traditional perioperative treatment of patients undergoing emergency surgery is often a non-standardized practice [8]. Several specific conditions may warrant a customized approach, including the presence of sepsis and infection, contamination at local sites and the type of intervention done or planned for the specific condition at hand. The common major emergency procedures and their consequences represent a massive healthcare burden, and there is tremendous potential for quality improvement [9].

Some recent data support the use of artificial intelligence (AI) to develop better clinical decision support tools based on machine learning [10]. Some studies tried to move from patient-controlled analgesia to AI-assisted patient-controlled analgesia [11].

The aim of the present guidelines is to suggest the appropriate pain management after non-traumatic emergency surgical intervention and to give to surgeons and physicians working in different settings a list of indications in order to prescribe the best analgesia possible in the absence of a multidisciplinary pain team. Four international recognized scientific societies: World Society of Emergency Surgery (WSES), Global Alliance for Infection in Surgery (GAIS), Italian Society of Anesthesia, Analgesia Intensive Care (SIAARTI), and American Association for the Surgery of Trauma (AAST), participated in the project and approved the final manuscript.

## Materials and methods

**Fig. 1:**
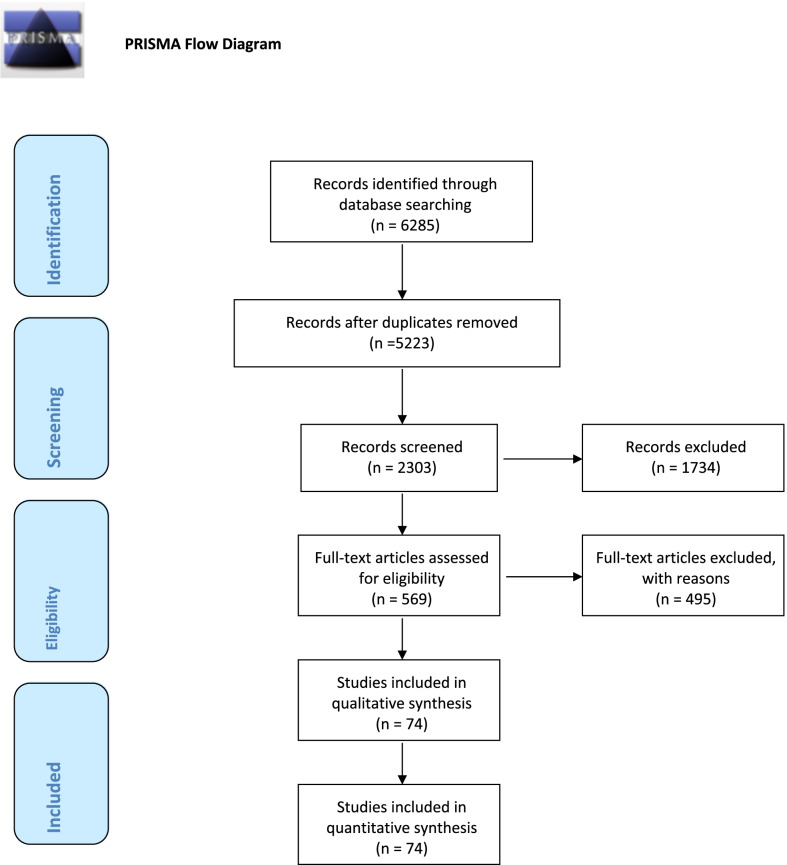
PRISMA flowchart

The bibliographer conducted a computerized search in different databases (MEDLINE, PubMed, Scopus, Web of Science, Embase). Citations were included up to June 2021 using the primary search strategy: acute postoperative pain, emergency surgery, pain assessment, acetaminophen, NSAIDs, ketamine, opioids, epidural anesthesia, peripheral nerve blocks, continuous wound infusion, local infiltration combined with AND/OR with synonyms and MeSH terms. We considered acute pain management after major abdominal pathology requiring urgent emergency laparotomy or laparoscopy, including reoperations after elective gastrointestinal surgery and reoperations after previous abdominal surgery. No language restriction was imposed. Duplicates and animal studies were removed. The dates were selected to allow comprehensive published abstracts of clinical trials, consensus conferences, comparative studies, congresses, guidelines, government publication, multicenter studies, systematic reviews, meta-analysis, large case series, original articles, and randomized controlled trials. Narrative review articles were also analyzed to identify other studies. Abstracts were screened, and irrelevant studies were removed; then, a full-text assessment of the articles was performed. Case reports were excluded. In case of disagreement between the two reviewers (FC and FCo), the consensus was reached by discussion. If there was no consensus, another two reviewers were sought (FCa and FFo). PRISMA guidelines flowchart [12] is reported in Fig. 1. Level of evidence (LoE) was graded in high, moderate, low, and very low. The grade of recommendation (GoR) graded as strong, moderate, and weak was calculated, keeping into consideration the GRADE model [13]. An international expert panel discussed the different issues in subsequent rounds. At each round, the manuscript was revised and improved. The final version about which agreement was reached resulted in the present manuscript.

### Notes on the use of the guideline

These guidelines present evidence-based methods for optimal management of acute postoperative pain management in emergency general surgery patients. The practice indications which were promulgated in this work do not represent a standard of practice. These are suggested plans of care based on the best available evidence and experts’ consensus, but they do not exclude other approaches as being within the standard of practice. For example, they should not be used to compel adherence to a given medical management method, which method should be finally determined by the treating health care provider after considering the conditions at the relevant medical institution (staff levels, experience, equipment, etc.) and the characteristics of the individual patient. However, the treatment results’ responsibility rests with those directly engaged and not with the consensus group.

#### Pain assessment and management

##### Statements

- Postoperative pain must be recognized and treated as soon as possible and as best as possible in all patients (high recommendation, intermediate quality evidence).
- Emergency general surgery may be associated with more severe postoperative pain; specific attention should be given to this patient group (GoR 2, LoE B) (moderate recommendation, intermediate quality evidence).
- Postoperative pain assessment, at rest and—if possible—on movement, is strongly recommended, to improve patient management after emergency surgery (moderate recommendation, intermediate quality evidence).
- Preemptive analgesia is a viable option in reducing postoperative opioid consumption (GoR 2, LoE B) (moderate recommendation, intermediate quality evidence).
- Adequate education for the patient and, if possible, of the family about the surgical and anesthesiologic treatment, options, plan, and aims of pain management should be performed whenever it is possible (GoR 1, LoE B) (high recommendation, intermediate quality evidence).
- Perioperative pain management should be implemented considering patient history, comorbidities, ongoing chronic therapy, and potential risk for substance abuse (GoR 1, LoE B) (high recommendation, intermediate quality evidence).
- Validated pain scales should be included into treatment planning, ongoing evaluation, and adjusting process (GoR 1, LoE B) (high recommendation, intermediate quality evidence).
- Pain management should be adjusted to ensure the greatest effect and the lowest side effects possible (GoR 1, LoE B) (high recommendation, intermediate quality evidence).

Emergency general surgery (EGS) is related to a worse acute postoperative pain compared to elective surgery [14–18]. Patient- and family-centered education is regarded as very important during the preoperative and postoperative periods [14, 15, 17, 19–22]. Telling patients how a drug is chosen, its properties and effects, understanding its side effects, and shared in the decisions helps reduce APP. A recent study proved that a lower educational level worsens pain [23].

An accurate evaluation including psychiatric comorbidities, namely dementia and delirium, may facilitate APP management since pain assessment techniques in these conditions may be much more time-consuming [19, 21, 24]. Assessment tools that incorporate a behavioral component for pain scoring have demonstrated validity in patients with dementia [25]. Special attention should be paid to the treatment of anxiety [26], evaluation of depression [26], and catastrophizing [15, 17]. Uncontrolled pain syndrome is manifested by tachycardia, arterial hypertension, increased rigidity of the muscles of the anterior abdominal wall and chest muscles, which leads to alteration of the ventilation and hypoxemia, difficulties in coughing and definitively to an increased risk of respiratory infectious complications. Enhanced sympathetic stimulation inhibits peristalsis and at the same time increases the tone of the smooth muscles of the intestine, which is fraught with the development of postoperative paresis. In addition, postoperative pain syndrome prevents early mobilization of patients and also contributes to their emotional and physical suffering, sleep disturbances. A sudden increase in pain, especially associated with the appearance of tachycardia, hypotension, hyperthermia, requires an urgent comprehensive assessment of the patient’s condition, since this may be a harbinger of postoperative complications (bleeding, anastomotic leaks, deep vein thrombosis, etc.).

All these conditions may contribute to worsening APP outcome. Coping strategies could be used specially to contrast catastrophizing thoughts.

The assessment of preoperative chronic pain is necessary because it is demonstrated that previous chronic pain history since may be associated to a worse APP [22]. Moreover, high APP pain level may lead to persistent postoperative pain [16, 17, 19, 20, 22, 27].

Accurate APP’s assessment, which is essential, is usually underestimated, underevaluated, and underperformed [14–16, 18, 20, 21, 23, 24, 28–32]. Some tools such as visual analog scale (VAS) score for some aspects seem to be inappropriate in measuring APP since it does not give a multidimensional pain evaluation. Hence, predictor index or questionnaire should be adopted to give the best APP evaluation.

Consideration should be given to APP follow-up evaluation and adequate therapy during the postoperative period. In fact, pain drugs are often administered not at regular intervals [18, 33] nor according to pain scales [15, 18, 33]. Local policy must include standard interval at which a patient is assessed and reassessed for pain. After a pain intervention is completed, reassess patients for both pain control and adverse reactions to the intervention at an appropriate interval based on the anticipated effect. When a significant change in worsening pain level is reported, reevaluate the patient for possible postoperative complications. A combined nurse service with clinician supervision seems to provide better outcomes in APP management [14, 15, 18, 20, 21, 23, 24, 34, 35]. It is proven that 24 h/day monitoring with a regular assessment/documentation guarantees a better pain treatment [14, 20].

In addition to psychiatric comorbidities, chronic pain, and patient dealing with substances of abuse, special attention should be paid to OSAS patients, since a correlation with APP is not fully understood [36, 37]. In this category of patients, antalgic therapy recommendation aims to reduce as much as possible the use of opioids to prevent possible cardiopulmonary complications. Furthermore, literature advises to be aware of age, body mass index (BMI) and gender: Younger age [16, 18, 23] and female gender [16, 18, 33] could be risk factors for APP. Some studies show that low BMI is associated with better pain outcomes [37]. Also knowing about the patient’s smoking habits could improve APP [23].

Preemptive and preventive NSAIDs seem to reduce both pain and morphine use. Clinically significant adverse events from nonsteroidal anti-inflammatory drugs (NSAIDs) administered before surgery are possibly under-reported; for this reason, it is impossible to define with high level of evidence the safety of either preemptive or preventive NSAIDs [38].

#### Non-opioids drugs

##### Statements

- Opiates usage should be reduced as much as possible in postoperative pain management strategies (strong recommendation, intermediate quality evidence).
- Multimodal pain management should always be considered to improve analgesia while reducing individual class-related side effects; a pharmacological step-up approach including major opiates when necessary should be adopted (strong recommendation, intermediate quality evidence).
- Whenever contraindications are absent, acetaminophen, NSAIDs (strong recommendation, high-quality evidence), and gabapentinoids administration (moderate recommendation, moderate quality evidence) are recommended in multimodal analgesia.
- Acetaminophen administered at the beginning of postoperative analgesia may be better and safer than other drugs (strong recommendation, intermediate quality evidence).
- Acetaminophen used in multimodal and preemptive therapy is associated with a reduction of opiates side effects and improved postoperative outcomes (strong recommendation, intermediate quality evidence).
- Coxib administration may be considered if there are no contraindications (strong recommendation, moderate quality evidence).

Multimodal analgesia involves the use of different classes of analgesic medications (NSAIDs, COX2 inhibitors, gabapentinoids, or acetaminophen in combination with morphine IV-PCA) with different mechanisms of action on the peripheral and/or central nervous system.

The different combinations of these drugs lead to additive or synergistic effects on pain relief and can potentially reduce the side effects of mono-modal interventions. The drugs used for this purpose include:

- Acetaminophen (paracetamol): it is effective as an analgesic mainly if used in combination with NSAIDs or morphine. Its use reduces opioids use [39–41]
- NSAIDs: are indicated for the treatment of moderate pain when used alone. Their use in multimodal analgesia reduces morphine consumption and related side effects [40]
- Opiates: are the first-line therapy to treat pain in these patients. They also reduce anxiety and dyspnea [42, 43]. PCA is recommended when iv route is needed in patients with adequate cognitive functions, starting with bolus injection in opioid naïve patients [7].
- Gabapentinoids such as gabapentin and pregabalin can be considered as a component in multimodal analgesia. They act by decreasing the release of neurotransmitters in the synapse, thus providing a nociceptive blocking activity.
- Alpha-2-agonists: in addition to their anti-hypertensive effect, they have been shown to have a sympatholytic effect by inhibiting norepinephrine release, thus reducing the opiates requirements.

*Acetaminophen* in a multimodal regimen is a valid and effective option. A study conducted in nearly 800,000 patients undergoing common major surgical elective and emergency procedures showed that this drug in a multimodal therapy regimen provides a cost-effective strategy to improve outcomes and patient satisfaction with a side-effect profile that is superior to opioids alone in moderate–severe APP [44]. The use of acetaminophen is associated with shorter length of stay, decrease in opioid-related complication rates, and lower costs in a heterogeneous population of patient who underwent to elective and emergency cardiovascular, colorectal, general, obstetrics and gynecology, orthopedics, or spine surgery [45]. A case–control cohort study of 1231 patients undergoing gynecologic and abdominal surgery showed that ibuprofen and acetaminophen (600 mg every 6 h and 500 mg every 6 h) could offer an adequate postoperative pain control with a supply of opioids (hydrocodone or oxycodone) if needed [46]. A single study suggests the use of IV acetaminophen in the beginning of postoperative analgesia since its antalgic properties are better and safer than IV tramadol in patients undergoing laparoscopic cholecystectomy [47].

Different efficacy can be assessed according to the timing in the administration of acetaminophen, in the context of multimodal analgesia, as a preemptive analgesia. Acetaminophen used in multimodal and preemptive therapy (1 g before laparotomy with naproxen 250 mg and pregabalin 150 mg) was associated with a reduction of opiate side effects as well as a fewer length of stay, lower opioid-related complication rates, and lower costs compared to patients who had not received this treatment [48].

Intravenous acetaminophen (i.v. acetaminophen every 6 h from 6 h after surgery up to 72 h) can be associated with thoracic epidural anesthesia (TEA); a study has been shown to provide a superior postoperative pain management compared to TEA alone in a randomized controlled trial with 120 patients who underwent distal gastrectomy [49].

Caution is needed in the frail patient, especially in the context of coexisting liver disease. For an amount of acetaminophen infusion sufficient to ensure a significant reduction in postoperative pain compared with groups without treatment (*P* = 0.008), an increase in alanine aminotransferase has been observed (*P* = 0.043) [50].

*Perioperative NSAIDs* utilization results regarding the reduction of hospital stay and lowering morbidity have been demonstrated in elective surgery [51]. The literature suggests a potential correlation with dehiscence, technical failures, and wound healing inhibition in emergency general surgery patients with colon or rectal anastomoses [52]. There is not enough evidence to establish the effectiveness of NSAIDs beyond their safety profile.

In terms of efficacy for the individual NSAID drugs, no direct comparisons trial is available. Effectiveness analysis was conducted on the single NSAID once at a time. In the case of abdominal emergency surgery, it was found that perioperative administration of Ibuprofen IV 800 mg every 6 h decreased morphine requirements and pain score and it has been found safe and well tolerated [53]. The literature might suggest also considering the use of HPβCD-diclofenac in a multimodal approach to analgesia. HPβCD-diclofenac in postoperative setting reduces postoperative opioid requirements during the whole postoperative course (all *P* < 0.005 vs placebo) [54]. The combination of NSAIDs with acetaminophen improves the quality of pain relief compared to the appointment of each of the drugs separately [55].***.***

The use of coxib is effectiveness in a major surgery context [56, 57] since it provides analgesia and opioid-sparing effects in the 2–3 days immediately following major gastrointestinal surgeries employing laparotomy and reduces the VAS scores both at rest and with movement, reducing also opioids adverse effects in patients following liver resection. A word of caution must be spent regarding the associated use of coxib and NSAIDs as their combination seems to increase the incidence of myocardial infarction and to affect kidney function [58, 59]***.***

Results about the use of *gabapentinoids* in postoperative management in EGS are heterogeneous and conflicting [60].

Preemptive anesthesia with other medications such as gabapentinoids to treat postoperative pain could lower opioid consumption and pain scores.

Due to the paucity of the literature, it is not possible to provide specific indications for the use of *ketamine* in emergency abdominal surgery. According to the literature, a single dose or infusion of ketamine appears to reduce pain score, and opioid consumption in the 48 h following surgery, especially in patients who have undergone major chest, abdominal, and orthopedic surgery [61–63]. Evidence is reported from various types of surgery, including abdominal surgery, on the administration of ketamine added in an opioid intravenous patient-controlled analgesia (IV-PCA) with a reduction in pain, opiate consumption, and PONV up to 24–72 h after surgery [64, 65]. Ketamine is recommended in severe pain management, and subanesthetic doses considered to have evidence of efficacy in acute pain are boluses < 0.35 mg/kg and infusions at 0.5-1 mg/kg/h, with no intensive monitoring required [61]. The recommended dose in severe APP management using an IV-PCA is 1–5 mg. It should not be used in uncontrolled cardiovascular disease, pregnancy, active psychosis, severe liver dysfunction, high intracranial, and ocular pressure [61]. A prospective cohort study shows that perioperative adding of ketamine IV (0.25 mg/kg bolus followed by 0.25 mg/kg/h, maximum 1 mg/kg) to opioids did not improve postoperative pain after colorectal surgery perception nor decrease morphine equivalents maybe due to an inappropriate dose compared to bupivacaine 0.0625% and fentanyl 2 µg/ml [66].

The use of dexmedetomidine in major abdominal surgery can be considered. Dexmedetomidine in combination with fentanyl-based intravenous PCA (dexmedetomidine infusion rate: 0.07 μg/kg/h with a bolus dose of 0.007 μg/kg, and fentanyl infusion rate: 0.3 μg/kg/h with a bolus dose of 0.03 μg/kg allowed every 15-min lockout time) had the same antalgic effects of patient-controlled epidural analgesia (PCEA) without hemodynamic instability and with a less invasive technique [67]. However, no precise indication can be given for patients undergoing emergency abdominal surgery.

#### Opioid drugs

##### Statement

- In the treatment of moderate-to-severe pain, unresponsive to other medications and in which regional anesthesia techniques are not indicated, the use of major opiate is indicated (strong recommendation, moderate quality evidence).
- Initial infusion of opioids using intravenous patient-controlled analgesia should be avoided in opioid naïve patients (strong recommendation, moderate quality evidence).
- Sedation levels, respiratory status, and the possible development of adverse events in patients on systemic treatment with opioids must be regularly assessed (strong recommendation, weak quality evidence).
- If indicated, infusion of opiates using intravenous patient-controlled analgesia should be preferred to spinal patient-controlled analgesia in postoperative pain management whenever the intravenous route is viable (strong recommendation, moderate quality evidence).

Opioids are strong and fast acting analgesics that are very effective and convenient in use for severe APP. Unfortunately, opioids are also associated with an important side effect and the risk of drug dependency. We are now facing a worldwide opioid crisis that causes 22,000 deaths annually in the USA alone. Although opioids have a central place in management of severe APP, it is therefore important to select an appropriate opioid for a time period as short as possible.

The use of PCA with major opiates after EGS is effective and useful [68]. Although the literature confirms the superiority of treatment for severe APP with opiates PCA, there is no clear evidence about which opiate drug should be preferred. Morphine, which is by far the most widely used drug, is not the ideal molecule as it has high renal clearance with potential accumulation and adverse effects [69, 70]. The alternatives are fentanyl, oxycodone, and sufentanil. As regards the type of molecule to be used, the literature does not exclusively address an opiate: Oxycodone (0.7 mg/kg—background continuous infusion of 1 to 2 mL/h 1 mL bolus with a 15-min lockout) is comparable to fentanyl (fentanyl 12 mg/kg,—background continuous infusion of 1 to 2 mL/h 1 mL bolus with a 15-min lockout) in the relief of postoperative pain following laparotomy. Oxycodone only seems to provide a slightly better postoperative pain relief and less sedation, but it is also associated with more side effects than fentanyl [71]. No significant pain scores differences at 5 and 30 min postoperatively were registered between oxycodone and fentanyl treatment in patients undergoing laparoscopic cholecystectomy [72]. The use of sublingual sufentanil tablet system has been compared to PCA. Sufentanil seems to be an appropriate choice due to its high affinity for the μ opioid receptor, its high therapeutic index, and the absence of clinically relevant active metabolites [4]. The sublingual sufentanil tablets (SSTs) is a noninvasive combination of a drug and a medical device which contains a cartridge of 40 tablets (sufentanil 15 µg) with a lockout interval of 20 min that seems to have better safety and tolerability in patients with open abdominal surgery or major orthopedic surgery [73]. In open abdominal surgery, SST 30mcg is effective for the management of moderate-to-severe postoperative pain [74]. The literature regarding SST administration in EGS is scarce, so no definitive indication can be given.

In some context, opiates are rarely given during the postoperative period to treat moderate-to-severe acute pain (13%, while other analgesics were administered in the 86.4%) [75]. However, in perioperative conditions of moderate-to-severe pain unresponsive to other treatment opioids represent a viable and effective option. Side effects of opioid analgesics are dose-dependent, and at high doses, they can induce hyperalgesia [76]***.***

Whenever PCA is not available or cannot be administered due to due to clinical or social barriers, transdermal fentanyl patch (25 μg/h) may be used. These patches should be affixed 12–14 h before surgery and avoid the continuous IV infusion of fentanyl after surgery. Transdermal administration is shown to reach a higher constant concentration without any evidence of respiratory depression [77]. There are no differences in pain score between transdermal fentanyl patch and IV fentanyl and the use of rescue analgesics after laparoscopic cholecystectomy [77].

#### Route of drugs administration

##### Statement

- Oral administration of analgesic drugs should be preferred over intravenous route whenever feasible, and drugs absorption may be reasonably warranted (strong recommendation, moderate quality evidence).
- The intramuscular route should be avoided in postoperative pain management (strong recommendation, moderate quality evidence).
- Epidural and regional anesthesia is recommended in emergency general surgery, whenever feasible and if not delaying the emergency procedures (intermediate recommendation, intermediate quality evidence).
- Neuraxial administration of magnesium, benzodiazepines, neostigmine, tramadol, and ketamine should be avoided (strong recommendation, moderate quality evidence).
- Patients with neuraxial anesthesia must be monitored and assessed adequately (strong recommendation, low quality evidence).

Available evidence suggests that intravenous administration of opioids or NSAIDs is not superior for postoperative analgesia compared with oral administration. Emergency abdominal surgeries usually affect the ability to take medications orally or enterally. Moreover, drug absorption after oral administration is a highly complex process, depending on both physicochemical properties of the drug and physiological conditions of the body. Postoperative ileus is an inevitable consequence of abdominal surgery caused by pharmacological agents (anesthetics, opioids) in the perioperative period, neural mechanisms, and intestinal inflammation due to the manipulation during the surgery—which is the most important pathophysiological mechanism. In emergency conditions, inflammatory cascade of events leads to a higher inflammatory background. [15].

Furthermore, drugs are absorbed in unionized state, which is dependent upon GI pH; also, the changing of gastric emptying rate and intestinal transit time can affect drug absorption. The perioperative period after major abdominal surgeries is characterized by a slower gastric emptying rate with a higher risk of aspiration and an impaired intestinal transit time. For these reasons, the oral route of administration is frequently not suitable in the acute postoperative.

Oral medication in an acute postoperative setting could be administered before surgery—in a preventive way—or in the postoperative period through the sublingual route.

PCA showed a better pain relief in abdominal surgery compared to intravenous morphine continuous infusion (2 mg/h as basal infusion and 3–5 mg IV bolus administration every time when required to obtain NRS below 3/10) [78]. Patient-controlled analgesia (PCA) either intravenous or epidural provides superior postoperative pain control and patient satisfaction, even if it increased amount of opioid consumption [79].

A study has tried to confirm this by correlating EA and reduced complications after colectomy and support a possible role for epidural analgesia in a multimodal analgesic regimen after open colectomy [80]. Emergency colorectal resection for colorectal cancer in colonic obstruction without peritonitis and in patients with elective surgical intervention can be considered similar. For this reason, the approach in pain management can be mutualized from literature evidence obtained in elective patients. One main point to be considered is the cautious usage of opiates in those patients who may present dynamical ileus due to intestinal overdistension. These patients may have difficulties in recovering intestinal motility, and opiates may exacerbate the ileus.

Thoracic epidural analgesia (TEA) use has been associated with a lower incidence of paralytic ileus, attenuation of the surgical stress response, improved intestinal blood flow, improved analgesia, and reduction of opioid use [81]. PCEA is suggested in fragile patients because this approach would seem to decrease stress response and minimize immune dysfunction improving plasma cortisol (Cor), interleukin (IL)-6 and IL-17 levels, and helper T-cell differentiation in esophageal carcinoma patients. PCEA stress response effects are most pronounced upon combination TEA/PCEA treatment [82]. On the other hand, in the elderly patient, as in the young patient, this type of analgesia was associated with more frequent episodes of numbness and motor weakness/deficits, hypotension, and nausea/vomiting comparing it to morphine PCA. Therefore, a retrospective analysis suggests using different PCEA strategies of administration regimens or adverse effects prevention for young and elderly patients [83]. We also suggest caution in males who underwent EA because of the possibility of urinary retention which slows patient recovery and may impair renal function. Urinary retention after EA had a higher incidence, and routine transurethral bladder drainage with early removal to prevent urinary tract infection is suggested [84].

TEA for pain management in APP seems to be useful also in emergency major abdominal surgery. The most part of the literature showed benefits in oncological elective surgery [51, 85, 86].

TEA does not exclude a multimodal approach, for example, in combination with intravenous acetaminophen. It seems to provide a superior postoperative pain management compared with TEA alone [49]. The type of drug infused and its concentration to provide a differential sensory block with the same effectiveness have been evaluated. Both epidural infusions of 0.125% ropivacaine with 1 μg/ml fentanyl and 0.125% bupivacaine with 1 μg/ml fentanyl in major abdominal surgery showed the same antalgic effect with minimal motor block [87]. A prospective randomized study showed that ropivacaine with nalbuphine is more effective than ropivacaine with butorphanol for immediate postoperative pain relief in patients undergoing emergency exploratory laparotomy [88].

#### Perioperative nerve block and local infiltration

##### Statement

- Regional anesthesia techniques are effective in both adults and children in site-specific surgery (strong recommendation, high-quality evidence).
- Abdominal wall blocks can be considered a technique with an opioid-sparing effect (intermediate recommendation, intermediate quality evidence).
- Transversus abdominal plane (TAP) block in patients undergoing laparoscopic abdominal surgery is proved to be a safe and effective method to treat postoperative pain (intermediate recommendation, intermediate quality evidence); a rectus sheath block is a viable alternative to the TAP block (intermediate recommendation, intermediate quality evidence).
- Local wound infusion is suggested as a component of multimodal analgesia (weak recommendation, moderate quality evidence).
- The use of continuous local wound infusion catheters is associated with a significant decrease in visual analogue scores for pain at rest and with activity (weak recommendation, intermediate quality evidence).
- Use of continuous local wound infusion catheters consistently reduces the need for opioids, both as rescue and total dose (weak recommendation, intermediate quality evidence).
- A pre-peritoneal catheter is not associated with an increased risk of surgical site infection (weak recommendation, intermediate quality evidence).
- Pre-peritoneal catheters must have a planned removal process including institution of appropriate analgesia (moderate recommendation, intermediate quality evidence).

The use of perineural/local analgesia techniques is indicated in case of major interventions characterized by moderate-to-severe pain (NRS > 6) affecting the chest and abdominal wall [89]. The most frequently used peripheral nerves block (PNB) is the use of the rectus sheath block and the transversus abdominis plane (TAP) block. Regarding the timing of the block, it is suggested to perform rectus sheath block before surgery in pain management for laparoscopic abdominal surgery [90].

TAP block in patients undergoing laparoscopic abdominal surgery is proved to be a safe and effective in treating postoperative pain with a statistically significant decrease in VAS at 12 h after the surgery [91, 92].

As far as the use of adjuvants is concerned, there is evidence in the literature suggesting the use of perineural dexamethasone with ropivacaine for thoracic paravertebral block in patient undergoing thoracotomy since it improves postoperative analgesia quality. NRS scores, rate of analgesic usage, ambulation time, and intestinal function recovery time were significantly reduced in patients with local wound infusion (LWI) compared to placebo at each postoperative time point (6, 12, 24 and 48 h; *P* < 0.05). And the NRS scores of patients with LWI at 12 h post-surgery were significantly reduced compared with the PCA group (*P* < 0.05) [93, 94].

Local anesthetics would directly block transmission of pain from nociceptive afferents from the wound surface; local anesthetic may also inhibit local inflammatory response to injury [95].

### Postoperative pain management associated constipation

Constipation does not affect everyone who has surgery, but it is a relatively common side effect of pain medications, anesthesia, and a lack of mobility. The anesthetic regimen administered during surgery is likely to have an effect on constipation during recovery. Both the type of anesthesia and the surgical duration affect the likelihood of postoperative constipation. Surgeries that last longer in duration tend to be associated with a higher predisposition to constipation. Post-surgery constipation is often a result of opioid pain medications; given either as part of the anesthesia or for pain relief following the surgery, opioid-induced constipation (OIC) in patients receiving opioids is persistent and the most frequently reported side effect [96, 97].

Multimodal analgesia combines regional analgesia, non-opioid analgesics [acetaminophen, nonsteroidal anti-inflammatory drug (NSAID) or cyclooxygenase (COX)-2 specific inhibitor], lidocaine infusions, gabapentinoids, and ketamine. Numerous studies have shown the opioid-sparing effect of this approach resulted in an accelerated GI recovery and improved outcomes. However, an optimal combination of these elements has not yet been elucidated [98, 99].

Caffeine is widely known as a stimulant to colonic motor activity in animals and humans. Clinical trials have shown that caffeinated drinks decrease the time to flatus and first bowel movement and if given as soon as 2 h after surgery, it may accelerate GI recovery and reduce LOS [100, 101].

Lastly, the use of regional anesthesia over general anesthesia whenever possible may help in reducing the number of drugs used and thus may reduce the likelihood of constipation after surgery.

## Patients not amenable for interventions or already operated but not suitable for further interventions to manage affecting disease or complications

Unlike patients with treatable conditions or where active organ support is chosen for their care, there remains a group of frail patients or those for whom surgical intervention would be non-beneficial and there is a defined role for analog-sedative medications in the treatment of this cohort.

### Pain assessment

- Periodic assessment of pain score is mandatory using validated systems to evaluate the response to treatments and to allow adjustments (strong recommendation, intermediate quality evidence).
- Observational pain scales are less reliable than patient reported metrics. However, in the non-communicative patient, observational pain scales should still be applied (strong recommendation, low quality evidence)

Different pain assessment tools have been validated: NRS (Numeric Rating Scale), VAS (visual analog scale) and VRS (Verbal Rating Scale) or the Behavioral Pain Scale (BPS) and the Critical Care Pain Observation Tool (CCPOT) in case of critically ill patients. There is no evidence of the superiority of one specific tool, and the choice should be made according to patient status (developmental, cognitive, educational, cultural status, and language differences). It remains that patient self-assessment of pain is the most valuable tool. Patient’s opinion must be listened and his/her feelings trusted [102–104].

### Drug therapy

- Multimodal analgesia is suggested to treat moderate-to-severe pain in patients not amenable for surgical interventions or already operated on but not suitable for further interventions (strong recommendation, intermediate quality evidence).
- The combination of systemic multimodal analgesia with regional analgesia is suggested in patients already operated on but not suitable for further interventions (strong recommendation, intermediate quality evidence)
- Patients not amenable for surgical interventions or already operated on but not suitable for further interventions should be considered for palliation to achieve the control of all other related symptoms such as nausea, vomiting, dyspnea, agitation, and delirium (strong recommendation, intermediate quality evidence)

In patients not suitable for further intervention due to the clinical conditions or due to the disease itself, multimodal analgesia is effective and should be adopted in postoperative pain management. The different combinations of the known drugs with their additive or synergistic effects on pain relieve reduce the side effects of mono-modal interventions and increase the effect on pain reduction (Table 1).

**Table 1:** Initial opioids titration in opioid-naive patients during palliate care

Drug	Frequency	Intravenous or subcutaneous	Oral
Morphine	8/24 h	2.5–10 mg	2.5–10 mg
Fentanyl	8/24 h	25–100 mcg	Not Available

### Nausea and vomiting

- Nausea and vomiting should be managed with medications that target dopaminergic pathways (i.e., haloperidol, risperidone, metoclopramide, prochlorperazine) (high recommendation, intermediate quality evidence).
- Octreotide should be utilized in the treatment of nausea and vomiting due to bowel obstruction caused by cancer (high recommendation, intermediate quality evidence).
- We suggest adding a second agent (i.e., ondansetron) to control nausea and vomiting when the first-line medications are unable to control the symptoms (high recommendation, intermediate quality evidence).

Aforementioned drugs are routinely used as therapies for nausea because of their inhibition of receptors in the brain’s chemoreceptor trigger zone. Studies have not shown newer 5-HT_3_ medications to be superior to older dopaminergic agents in treating nausea at the end of life [105–108].

All medications that may be added as second agent in treating refractory nausea are listed in Table 2 together with the setting of use and doses and route of administration.

**Table 2:** Additional agents for the treatment of nausea and vomiting

Drug	Setting	Frequency	Intravenous or subcutaneous	Oral	Topic
Scopolamine	Increased oral secretions	1/72 h	–	–	1.5–3 mg
Lorazepam	Anticipatory nausea	4/24 h	0.5–2 mcg	0.5–2 mcg	–
Dexamethasone	Bowel obstruction Intracranial hypertension	3–6/24 h	2–8 mg	2–8 mg	–
Haloperidol	Nausea	3–6/24 h	0.5–2 mg	0.5–2 mg	–
Prochlorperazine	Nausea	3–4/24 h	5–10 mg	5–10 mg	–
Chlorpromazine	Nausea	3–4/24 h	12.5–25 mg	25–50 mg	–

### Delirium

- Whenever appropriate, the evaluation of delirium should be done using standardized assessment tools validated in critically ill patients like Confusion Assessment Method for the Intensive Care Unit (CAM-ICU) or Intensive Care Delirium Screening Checklist (ICDSC) (high recommendation, intermediate quality evidence).
- Possible causes of delirium, including drug-induced delirium, must be minimized, and pain control should be optimized before the pharmacological approach is implemented (high recommendation, intermediate quality evidence).
- The use of i.v. haloperidol or droperidol in hyperactive (RASS + 1/ + 4) or hypoactive (RASS 0/-3) delirium with or without hallucinations is recommended (intermediate recommendation, intermediate quality evidence).

Delirium is common in the last weeks/days of life, so it must be considered in every patient in palliative care showing a change in behavior. Symptoms can affect different areas of cognition (memory, orientation, language, visuospatial ability, or perception) and may include hallucinations and disturbances in the sleep–wake cycle and can cause distress in both the patients experiencing it and those around them (DSM-5 [109]). Validated tools have been developed to allow screening of delirium to help non-specialists to address a diagnosis [110, 111].

Reversible causes account for 30–50% cases of delirium, especially drugs and poorly controlled pain. Drugs potentially responsible for delirium include benzodiazepines, corticosteroids, anticholinergics, opioids, and other drugs with psychoactive properties. Other possible reversible causes are metabolic disturbances (electrolyte imbalances, dehydration, hypo- or hyperglycemia), hypoxia, anemia, sepsis [112].

Overall, only 50% of delirium cases are reversible. The other half requires medical management which focuses on reducing agitation and perceptual abnormalities. Haloperidol is the drug of choice in the pharmacological treatment of delirium. An initial dose of 0,5-2 mg in. slow iv bolus may be used off-label. Haloperidol is associated with extrapyramidal side effects and lengthening of QT [110, 111].

### Dyspnea

- The assessment of respiratory distress should be done using a standardized assessment tool and the presence of suggestive objective signs. We recommend a stepwise approach to the treatment of dyspnea (high recommendation, intermediate quality evidence)
- When death is not imminent, the treatment of the etiology of dyspnea is recommended (high recommendation, intermediate quality evidence).
- Noninvasive ventilation to control dyspnea is suggested only until a properly deep sedation is reached or when sedation is inadequate. We recommend noninvasive ventilation only in predisposed settings with trained medical staff (high recommendation, intermediate quality evidence).
- Opioid usage as first-line treatment for dyspnea is recommended (high recommendation, intermediate quality evidence)

Dyspnea is the subjective awareness of altered respiratory function which usually results in respiratory distress. Respiratory distress is the totality of behavioral modifications that can be observed and measured (use of respiratory accessory muscles, nasal flaring, tachypnea, tachycardia, paradoxical breathing, fearful facial expression). Dyspnea is usually present in dying patients. When possible, being a subjective symptom, it must be assessed directly with the patient using standardized assessment tools [110].

The use of non-medical strategies to decrease respiratory distress should be considered like optimal positioning in sitting position, increased ambient air flows, use of fans, cold air. Noninvasive ventilation techniques as oxygen therapy include high-flow nasal cannula (HFNC) and continuous positive airway pressure (CPAP) ventilation to relief or mitigating dyspnea [110] by reducing the work of breathing in the absence of formal contraindication (intestinal occlusion and vomiting). Aspiration of airways if rattle is present. Anti-secretory medications may or may not be useful or required to decrease pulmonary secretions [113, 114].

Sedation with benzodiazepines or propofol can be considered as second line if dyspnea is not resolved with adequate doses of opioids since fear and anxiety can be con causes of the dyspnea in the dying patient [115].

## Conclusions

Dealing with acute postoperative pain in the emergency abdominal surgery setting is complex, requires special attention, and should be multidisciplinary. Several tools are available, and their combination is mandatory whenever is possible. Analgesic approach to the various situations and conditions should be patient based and tailored according to procedure, pathology, age, response, and available expertise. A better understanding of the patho-mechanisms of postoperative pain for short- and long-term outcomes is necessary to improve prophylactic and treatment strategies.

## Data Availability

Not applicable.

## Abbreviations

APP: Acute postoperative pain
i.v.: Intravenous
p.o.: Per oral
s.c.: Subcutaneous
EA: Epidural analgesia
PCA: Patient-controlled analgesia
CPNB: Continuous peripheral nerve blocks
EGS: Emergency general surgery
NSAIDs: Nonsteroidal anti-inflammatory drugs
TEA: Thoracic epidural anesthesia
PCEA: Patient-controlled epidural analgesia
TAP: Transversus abdominal plane
LWI: Local wound infusion
OIC: Opioid-induced constipation
CCPOT: Care Pain Observation Tool
HFNC: High-flow nasal cannula
CPAP: Continuous positive airway pressure
COX: Cyclooxygenase

## References

[1] Boyd-Carson H (2020). A review of surgical and peri-operative factors to consider in emergency laparotomy care. Anaesthesia.

[2] Jhanji S, Pearse RM (2009). The use of early intervention to prevent postoperative complications. Curr Opin Crit Care.

[3] Nilsson U, Gruen R, Myles PS (2020). Postoperative recovery: the importance of the team. Anaesthesia.

[4] Sacerdote P, Coluzzi F, Fanelli A (2016). Sublingual sufentanil, a new opportunity for the improvement of postoperative pain management in Italy. Eur Rev Med Pharmacol Sci.

[5] Meissner W (2015). Improving the management of post-operative acute pain: priorities for change. Curr Med Res Opin.

[6] Fishman SM., Bonica’s Management of Pain. Lippincott Williams & Wilkins; 2012. 701 p.

[7] Chou R, Al. E. Management of postoperative pain: A clinical practice guideline from the Ameri-can pain society, the American society of regional anesthesia and pain medicine, and the Ameri-can society of anesthesiologists’ committee on regional anesthesia. J Pain. 2016;17(2):131–57. 10.1016/j.jpain.2015.12.008 26827847

[8] Paduraru M, Ponchietti L, Casas IM, Svenningsen P, Zago M (2017). Enhanced Recovery after Emergency Surgery: A Systematic Review. Bull Emerg Trauma.

[9] Stewart B, Khanduri P, McCord C, Ohene‐Yeboah M, Uranues S, Rivera FV, et al. Global disease burden of conditions requiring emergency surgery. BJS (British J Surg. 2014;101(1):e9–22. 10.1002/bjs.9329 24272924

[10] Mcgrath H, Flanagan C, Zeng L, Lei Y (2019). Future of Artificial Intelligence in Anesthetics and Pain Management. J Biosci Med.

[11] Wang R, Wang S, Duan N, Wang Q (2020). From patient-controlled analgesia to artificial intelligence-assisted patient-controlled analgesia: practices and perspectives. Front Med.

[12] Page MJ, Moher D (2017). Evaluations of the uptake and impact of the Preferred Re-porting Items for Systematic reviews and Meta-Analyses (PRISMA) Statement and extensions: a scoping review. Syst Rev.

[13] Guyatt GH, Oxman AD, Kunz R, Vist GE, Falck-Ytter Y, Schünemann HJ; GRADE Working Group. What is “quality of evidence” and why is it important to clinicians? BMJ. 2008;336:995–998. 10.1136/bmj.39490.551019.BE PMC2364804 18456631

[14] Liu D, Ma J, Zhang Z, Yu A, Chen X, Feng C, et al. Management of postoperative pain in medical in-stitutions in Shandong Province in China. Medicine (Baltimore). 2016;12;95(6). 10.1097/MD.0000000000002690 PMC4753895 26871800

[15] Van Boekel RLM, Vissers KCP, van der Sande R, Bronkhorst E, Lerou JGC, Steegers MAH. Moving beyond pain scores: multidimensional pain assessment is essential for adequate pain manage-ment after surgery. PLoS One. 2017;12(5). 10.1371/journal.pone.0177345 PMC5425226 28489926

[16] Farić N, Barać I, Paarić S, Lovrić I, Ilakovac V (2017). Acute postoperative pain in trauma patients-the fifth vital sign. Open Access Maced J Med Sci.

[17] Wang Y, Liu Z, Chen S, Ye X, Xie W, Hu C (2018). Identifying at-risk subgroups for acute postsurgical pain: a classification tree analysis. Pain Med.

[18] Murray AA, Retief FW (2016). Acute postoperative pain in 1 231 patients at a developing country refer-ral hospital: Incidence and risk factors. South Afr J Anaesth Analg.

[19] Khatib SK, Razvi SS, Kulkarni SS, Parab S (2017). A multicentre survey of the current acute post-operative pain management practices in tertiary care teaching hospitals in Maharashtra. Indian J Anaesth.

[20] Montes A, Aguilar JL, Benito MC, Caba F, Margarit C (2017). Management of postoperative pain in Spain: a nationwide survey of practice. Acta Anaesthesiol Scand.

[21] Medrzycka-Dabrowka W, Dąbrowski S, Gutysz-Wojnicka A, Gawroska-Krzemińska A, Ozga D (2017). Barriers perceived by nurses in the optimal treatment of postoperative pain. Open Med.

[22] Polanco-Garcia M, Garcia-Lopez J, Fabregas N, Meissner W, Puig M (2017). Postoperative pain man-agement in spanish hospitals: a cohort study using the PAIN-OUT registry. J Pain.

[23] Peng LH, Jing JY, Qin PP, Su M (2016). A multi-centered cross-sectional study of disease burden of pain of inpatients in southwest China. Chin Med J (Engl).

[24] Mędrzycka-Dąbrowska WA, Dąbrowski S, Basiński A, Pilch D. Perception of barriers to postopera-tive pain management in elderly patients in Polish hospitals with and without a “Hospital With-out Pain” Certificate–a multi-center study. Arch Med Sci. 2016 A. 10.5114/aoms.2015.54768 PMC4947611 27478463

[25] Ferrari R, Martini M, Mondini S, Novello C, Palomba D, Scacco C (2009). Pain assessment in non-communicative patients: the Italian version of the Non-Communicative Patient’ s Pain Assessment Instrument ( NOPPAIN ). Aging Clin Exp Res.

[26] Bradshaw P, Hariharan S, Chen D (2016). Does preoperative psychological status of patients affect post-operative pain? A prospective study from the Caribbean. Br J Pain [Internet].

[27] Guimaraes-Pereira L, Valdoleiros I, Reis P, Abelha F. Evaluating persistent postoperative pain in one tertiary hospital: incidence, quality of life, associated factors, and treatment. Anesth Pain Med [Internet]. 2016 [cited 2019 Oct 31];6(2). 10.5812/aapm.36461 PMC4886451 27252908

[28] Cakir U, Cete Y, Yigit O, Bozdemir MN (2018). Improvement in physician pain perception with using pain scales. Eur J Trauma Emerg Surg [Internet].

[29] Chabowski M, Junke M, Juzwiszyn J, Milan M, Malinowski M, Janczak D (2017). Adaptation to illness in relation to pain perceived by patients after surgery. J Pain Res.

[30] Dragojevic-Simic V, Rancic N, Stamenkovic D, Simic R (2017). Utilization of parenteral morphine by ap-plication of ATC/DDD methodology: retrospective study in the referral teaching hospital. Front Public Health.

[31] Myles PS, Myles DB, Galagher W, Boyd D, Chew C, MacDonald N, et al. Measuring acute postoper-ative pain using the visual analog scale: the minimal clinically important difference and patient acceptable symptom state. Br J Anaesth. 2017;1. 10.1093/bja/aew466 28186223

[32] Ledowski T, Schneider M, Gruenewald M, Goyal RK, Teo SR, Hruby J (2019). Surgical pleth index: pro-spective validation of the score to predict moderate-to-severe postoperative pain. Br J Anaesth.

[33] Borys M, Zyzak K, Hanych A, Domagała M, Gałkin P, Gałaszkiewicz K (2018). Survey of postoperative pain control in different types of hospitals: a multicenter observational study. BMC Anesthesiol.

[34] Du C, Li H, Qu L, Li Y, Bao X (2019). Personalized nursing care improves psychological health, quality of life, and postoperative recovery of patients in the general surgery department. Int J Clin Exp Med.

[35] Haghighi MJ, Shahdadi H, Moghadam MP, Balouchi A. The Impact of Evidence-Based Practices on Postoperative Pain in Patients undergoing Gastrointestinal Surgery in Amiralmomenin Hospital in Zabol During 2014–2015. J Clin Diagn Res 2016 Jul;10(7):IC01–4. 10.7860/JCDR/2016/20961.8119 PMC5020264 27630865

[36] Strutz P., Aranake-Chrisinger A., Willingham M., Kronzer V.L., Abdallah A.B., Haroutounian S., et al. Obstructive sleep apnea as an independent predictor of postoperative delirium and pain: An observational study of a surgical cohort. Anesth Analg. 2018;1. 10.12688/f1000research.14061.1 PMC6039916 30026927

[37] Strutz PK (2019). The relationship between obstructive sleep apnoea and postoperative deliri-um and pain: an observational study of a surgical cohort. Anaesthesia.

[38] Doleman B, Leonardi-Bee J, Heinink TP, Boyd-Carson H, Carrick L, Mandalia R, Lund JN, Williams JP. Pre-emptive and preventive NSAIDs for postoperative pain in adults undergoing all types of surgery. Cochrane Database Syst Rev. 2021;6(6):CD012978. d. 10.1002/14651858.CD012978.pub2 PMC8203105 34125958

[39] Hyllested M, Jones S, Pedersen JL, Kehlet H (2002). Comparative effect of paracetamol, NSAIDs or their combination in postoperative pain management: a qualitative review. Br J Anaesth.

[40] Elia N, Lysakowski C, Tramè M (2005). Does multimodal analgesia with acetaminophen, nonsteroidal antiinflammatory drugs, or selective cyclooxygenase-2 inhibitors and patient-controlled analgesia morphine offer advantages over morphine alone?. Meta-Anal Randomized Trials Anesthesiol.

[41] McDaid C, Maund E, Rice S, Wright K, Jenkins B, Woolacott N. Paracetamol and selective and non-selective non-steroidal anti-inflammatory drugs (NSAIDs) for the reduction of morphine-related side effects after major surgery: a systematic review. In: NIHR Health Technology Assessment programme: Executive Summaries. 2003. 10.3310/hta14170 20346263

[42] Abernethy AP, Currow DC, Frith P, Fazekas BS, McHugh A, Bui C (2003). Randomised, double blind, placebo controlled crossover trial of sustained release morphine for the management of refractory dyspnoea. BMJ.

[43] Bruera E, MacEachern T, Ripamonti C, Hanson J (1993). Subcutaneous morphine for dyspnea in cancer patients. Ann Intern Med.

[44] Ladha, Karim S., et al. Variations in the use of perioperative multimodal analgesic therapy. Anes-thesiology J Am Soc Anesthesiol 124.4 (2016): 837–845. 10.1097/ALN.0000000000001034 PMC4792695 26835644

[45] Shaffer EE, Pham A, Woldman RL, Spiegelman A, Strassels SA, Wan GJ (2016). Estimating the effect of intravenous acetaminophen for postoperative pain management on length of stay and inpa-tient hospital costs. Adv Ther.

[46] Mark J, Argentieri DM, Gutierrez CA, Morrell K, Eng K, Hutson AD, et al. Ultrarestrictive opioid prescription protocol for pain management after gynecologic and abdominal surgery. JAMA Netw Open. 2018;1(8). 10.1001/jamanetworkopen.2018.5452 PMC6324564 30646274

[47] Bandey S, Singh V. Comparison between IV paracetamol and tramadol for postoperative analge-sia in patients undergoing laparoscopic cholecystectomy. J Clin Diagn Res. 2016;10(8):UC05–9. 10.7860/JCDR/2016/21021.8274 PMC5028584 27656532

[48] Amiri H, Mirzaei M, Pournaghi M, Fathi F. Three-agent preemptive analgesia, pregabalin-acetaminophen-naproxen, in laparotomy for cancer: a randomized clinical trial. Anesth Pain Med. 2016 Mar 15; 7(2). 10.5812/aapm.33269 PMC5556332 28824854

[49] Kinoshita J, Fushida S, Kaji M, Oyama K, Fujimoto D, Hirono Y, et al. A randomized controlled trial of postoperative intravenous acetaminophen plus thoracic epidural analgesia vs. thoracic epi-dural analgesia alone after gastrectomy for gastric cancer. Gastric Cancer. 2019;22(2):392–402. 10.1007/s10120-018-0863-5 PMC6394709 30088162

[50] Horita E, Takahashi Y, Takashima K, Saito K, Takashima Y, Munemoto Y (2018). Effectiveness of scheduled postoperative intravenous acetaminophen for colon cancer surgery pain. J Pharm Health Care Sci.

[51] Shida D, Tagawa K, Inada K, Nasu K, Seyama Y, Maeshiro T (2017). Modified enhanced recovery after surgery (ERAS) protocols for patients with obstructive colorectal cancer. BMC Surg.

[52] Haddad NN, Bruns BR, Enniss TM, Turay D, Sakran JV, Fathalizadeh A (2017). Perioperative use of nonsteroidal anti-inflammatory drugs and the risk of anastomotic failure in emergency general surgery. J Trauma Acute Care Surg.

[53] Gago Martínez A, Escontrela Rodriguez B, Planas Roca A, Martínez RA (2016). Intravenous Ibuprofen for treatment of post-operative pain: a multicenter, double blind, placebo-controlled, random-ized clinical trial. PLoS ONE.

[54] Gan TJ, Singla N, Daniels SE, Hamilton DA, Lacouture PG, Reyes CR (2016). Postoperative opioid sparing with injectable hydroxypropyl-β-cyclodextrin-diclofenac: pooled analysis of data from two Phase III clinical trials. J Pain Res.

[55] Ong CKS, Seymour RA, Lirk P, Merry AF (2010). Combining paracetamol (acetaminophen) with nonsteroidal antiinflammatory drugs: a qualitative systematic review of analgesic efficacy for acute postoperative pain. Anesth Analg.

[56] Essex MN, Xu H, Parsons B, Xie L, Li C (2017). Parecoxib relieves pain and has an opioid-sparing effect fol-lowing major gastrointestinal surgery. Int J Gen Med.

[57] Liu Y, Song X, Sun D, Wang J, Lan Y, Yang G (2018). Evaluation of intravenous parecoxib infusion pump of patient-controlled analgesia compared to fentanyl for postoperative pain management in laparoscopic liver resection. Med Sci Monit.

[58] Bhala N., Emberson J., Merhi A. et al. Vascular and upper gastrointestinal eff ects of non-steroidal anti-infl ammatory drugs: meta-analyses of individual. Lancet [Internet]. 2013;6736(13):1–11. 10.1016/S0140-6736(13)60900-9 PMC3778977 23726390

[59] Lee A, Mg C, Jc C, Jf K, Jp K. Effects of nonsteroidal anti-inflammatory drugs on postoperative renal function in adults with normal renal function ( Review ). Cochrane Database Syst Rev. 2009;(2). 10.1002/14651858.CD002765.pub3 PMC6516878 17443518

[60] Savard X, D M, Pinard A, Sc M. Perioperative use of gabapentinoids for the management of postoperative acute pain. Anesthesiology. 2020;(2):265–79. 10.1097/ALN.0000000000003428 32667154

[61] Laskowski K, Stirling A, McKay WP, Lim HJ (2011). A systematic review of intravenous ketamine for post-operative analgesia. Can J Anesth/J Can Anesth.

[62] Jouguelet-Lacoste J, La Colla L, Schilling D, Chelly JE (2015). The use of intravenous infusion or single dose of low-dose ketamine for postoperative analgesia: a review of the current literature. Pain Med.

[63] Wang L, Johnston B, Kaushal A, Cheng D, Zhu F, Martin J. Ketamine added to morphine or hydro-morphone patient-controlled analgesia for acute postoperative pain in adults: a systematic re-view and meta-analysis of randomized trials. Can J Anaesth. 2016 Mar. 10.1007/s12630-015-0551-4 26659198

[64] Assouline B, Tramèr M, Kreienbühl L, Elia N (2016). Benefit and harm of adding ketamine to an opioid in a patient-controlled analgesia device for the control of postoperative pain: systematic review and meta-analyses of randomized controlled trials with trial sequential analyses. Pain.

[65] Grass F, Cachemaille M, Martin D, Fournier N, Hahnloser D, Blanc C (2018). Pain perception after colorectal surgery: a propensity score matched prospective cohort study. Biosci Trends.

[66] Abdelgalil AS, Shoukry A, Kamel M, Heikal A, Ahmed N (2019). Analgesic potentials of preoperative oral pregabalin, intravenous magnesium sulfate, and their combination in acute postthoracotomy pain. Clin J Pain.

[67] Kim N, Kwon T, Bai S, Noh S, Hong J, Lee H (2017). Effects of dexmedetomidine in combination with fentanyl-based intravenous patient-controlled analgesia on pain attenuation after open gastrectomy in comparison with conventional thoracic epidural and fentanyl-based intravenous patient-controlled analgesia. Int J Med Sci.

[68] Hemmerling, Thomas M. Pain management in abdominal surgery. Langenbeck’s Archiv Surg 2018;403.7(2018):791–803. 10.1007/s00423-018-1705-y 30284029

[69] Gottschalk A, Ford J, Durieux ME, Tiouririne M (2010). Review article: the role of the perioperative period in recurrence after cancer surgery. Anesth Analg.

[70] Laulin J, Maurette P, Rivat C (2002). The role of ketamine in preventing fentanyl-induced hyperalgesia and subsequent acute morphine tolerance. Anesth Analg.

[71] Ding Z, Wang K, Wang B, Zhou N, Li H, Yan B (2016). Efficacy and tolerability of oxycodone versus fenta-nyl for intravenous patient-controlled analgesia after gastrointestinal laparotomy: a prospec-tive, randomized, double-blind study. Med.

[72] Choi EK, Kwon N, Park S-J. Comparison of the effects of oxycodone versus fentanyl on airway re-flex to tracheal extubation and postoperative pain during anesthesia recovery after laparoscopic cholecystectomy. Medicine (United States). 2018;97(13). 10.1097/MD.0000000000010156 PMC5895408 29595640

[73] Pogatzki-Zahn, Esther, et al. Real-world use of the sufentanil sublingual tablet system for patient-controlled management of acute postoperative pain: a prospective noninterventional study. Curr Med Res Opin 2020;36.2(2020):277–284. 10.1080/03007995.2019.1681133 31612723

[74] Hutchins JL, Leiman D, Minkowitz HS, Jove M, DiDonato KP, Palmer PP. An open-label study of sufentanil sublingual tablet 30 Mcg in patients with postoperative pain. Pain Med [Internet]. 2018;19(10):2058–68. 10.1093/pm/pnx248 PMC6176750 29126259

[75] García-Ramírez PE, González-Rodríguez SG, Soto-Acevedo F, Brito-Zurita OR, Cabello-Molina R, López-Morales CM (2018). Postoperative pain: frequency and management characterization. Co-lombian J Anesthesiol.

[76] Jarzyna D, Jungquist CR, Pasero C, Nisbet A, Dempsey SJ. American Society for pain management nursing guidelines on monitoring for opioid- induced sedation and respiratory depression. Pain Manag Nurs [Internet]. 2011;12(3):118–145.e10. 10.1016/j.pmn.2011.06.008 21893302

[77] Jang JS, Hwang SM, Kwon Y, Tark H, Kim YJ, Ryu BY, et al. Is the transdermal fentanyl patch an effi-cient way to achieve acute postoperative pain control? A randomized controlled trial. Medicine (United States). 2018;97(51). 10.1097/MD.0000000000013768 PMC6319953 30572528

[78] Zgâia A, Lisencu C, Rogobete A, Vlad C, Achimaş-Cadariu P, Lazăr G, et al. Improvement of recovery parameters using patient-controlled epidural analgesia after oncological surgery. A prospective, randomized single center study. Rom J Anaesth Intensive Care 2017;24(1)29–36. 2017;24(1):29–36. 10.21454/rjaic.7518.241.zga PMC5555424 28913495

[79] Regenbogen SE, Mullard AJ, Peters N, Brooks S, Englesbe MJ, Campbell DA (2016). Hospital analge-sia practices and patient-reported pain after colorectal resection. Ann Surg.

[80] Cummings K, Zimmerman N, Maheshwari K, Cooper G, Cummings L (2018). Epidural compared with non-epidural analgesia and cardiopulmonary complications after colectomy: a retrospective cohort study of 20,880 patients using a national quality database. J Clin Anesth.

[81] Helander EM, Webb MP, Bias M, Whang EE, Kaye AD, Urman RD (2017). Use of regional anesthesia tech-niques: analysis of institutional enhanced recovery after surgery protocols for colorectal sur-gery. J Laparoendosc Adv Surg Tech A.

[82] Gu CY (2015). Effects of epidural anesthesia and postoperative epidural analgesia on immune func-tion in esophageal carcinoma patients undergoing thoracic surgery. Mol Clin Oncol.

[83] Koh JC, Song Y, Kim SY, Park S, Ko SH, Han DW (2017). Postoperative pain and patient-controlled epidur-al analgesia-related adverse effects in young and elderly patients: a retrospective analysis of 2,435 patients. J Pain Res.

[84] Grass F, Slieker J, Frauche P, Solà J, Blanc C, Demartines N (2017). Postoperative urinary reten-tion in colorectal surgery within an enhanced recovery pathway. J Surg Res.

[85] Jeong O, Ryu SY, Park YK. Postoperative functional recovery after gastrectomy in patients un-dergoing enhanced recovery after surgery. Medicine (Baltimore). 2016;95(14). 10.1097/MD.0000000000003140 PMC4998752 27057836

[86] Forsmo HM, Erichsen C, Rasdal A, Körner H, Pfeffer F (2017). Enhanced recovery after colorectal surgery (ERAS) in elderly patients is feasible and achieves similar results as in younger patients. Gerontol Geriatr Med.

[87] Patil S, Kudalkar A, Tendolkar B. Comparison of continuous epidural infusion of 0.125% ropivacaine with 1 μg/ml fentanyl versus 0.125% bupivacaine with 1 μg/ml fentanyl for postoperative analgesia in major abdominal surgery. J Anaesthesiol Clin Pharmacol. 2018;34(1):29–34. 10.4103/joacp.JOACP_122_16 PMC5885444 29643619

[88] Babu S, Gupta BK, Gautam GK (2017). A comparative study for post operative analgesia in the emergen-cy laparotomies: thoracic epidural ropivacaine with nalbuphine and ropivacaine with butor-phanol. Anesth Essays Res.

[89] Fanelli A, Divella M, Compagnone C, Neurorianimazione SC, Gazzerro G, Marinangeli F, et al. Ge-stione e trattamento del Dolore Acuto Post-operatorio (DAP) [Internet]. [cited 2020 May 5] link: http://www.siaarti.it/SiteAssets/Ricerca/buona-pratica-clinica-.

[90] Jeong H-W, Kim CS, Choi KT, Jeong S-M, Kim D-H, Lee J-H. Preoperative versus postoperative rec-tus sheath block for acute postoperative pain relief after laparoscopic cholecystectomy: a ran-domized controlled study. J Clin Med. 2019;8(7). 10.3390/jcm8071018 PMC6679218 31336767

[91] El Sherif F, Mohamed S-B, Kamal S (2017). The effect of morphine added to bupivacaine in ultra-sound guided transversus abdominis plane (TAP) block for postoperative analgesia following lower abdominal cancer surgery, a randomized controlled study. J Clin Anesth.

[92] Guo J, Li H, Pei Q, Feng Z (2018). The analgesic efficacy of subcostal transversus abdominis plane block with Mercedes incision. BMC Anesthesiol.

[93] Lee SH, Sim W-S, Kim GE, Kim HC, Jun JH, Lee JY (2016). Randomized trial of subfascial infusion of ropivacaine for early recovery in laparoscopic colorectal cancer surgery. Korean J Anesthesiol.

[94] Wu Y-F, Li X-P, Yu Y-B, Chen L, Jiang C-B, Li D-Y (2018). Postoperative local incision analgesia for acute pain treatment in patients with hepatocellular carcinoma. Rev Assoc Med Bras.

[95] Hahnenkamp K, Theilmeier G, Van Aken HK, Hoenemann CW (2002). The effects of local anesthetics on perioperative coagulation, inflammation, and microcirculation. Anesth Analg.

[96] Müller-Lissner S, Bassotti G, Coffin B, Drewes A, Breivi H, Eisenberg E, et al. Opioid-Induced constipation and bowel dysfunction: a clinical guideline. Pain Med. 2016;1–27. 10.1093/pm/pnw255 PMC5914368 28034973

[97] Streicher JM, Bilsky EJ. Peripherally acting μ -opioid receptor antagonists for the treatment of opioid-related side effects: mechanism of action and clinical implications. J Pharm Pract. 2017; 10.1177/0897190017732263 PMC6291905 28946783

[98] Geltzeiler CB, Rotramel A, Wilson C, Deng L, Whiteford MH, Frankhouse J (2015). Prospective study of colorectal enhanced recovery after surgery in a community hospital. JAMA Surg.

[99] Agents P, Helander EM, Webb MP, Bias M (2017). A comparison of multimodal analgesic approaches in institutional enhanced recovery after surgery protocols for colorectal surgery. J Laparoendosc Adv Surg Tech.

[100] Kane TD, Tubog TD, Schmidt JR. The use of coffee to decrease the incidence of postoperative ileus: a systematic review and meta-analysis. J PeriAnesthesia Nurs [Internet]. 2019;(xxxx). Available from: 10.1016/j.jopan.2019.07.004 10.1016/j.jopan.2019.07.004 31859206

[101] Dulskas A, Klimovskij M, Vitkauskiene M. Effect of coffee on the length of postoperative ileus. Dis Colon Rectum. 2015;11(D):1064–9. 10.1097/DCR.0000000000000449 26445179

[102] Myles PS, Myles DB, Galagher W, Boyd D, Chew C, Macdonald N, et al. Measuring acute postoperative pain using the visual analog scale : the minimal clinically important difference and patient acceptable symptom state. Br J Anaesth [Internet]. 2017;118(3):424–9. Available from: http://dx.doi.org/10.1093/bja/aew466 10.1093/bja/aew466 28186223

[103] Mordecai L, Vindrola-padros C, Wood VJ, Swart N, Morris S, Williams A, et al. A novel inpatient complex pain team: protocol for a mixed-methods evaluation of a single-centre pilot study. BMJ Open. 2018;1–8. 10.1136/bmjopen-2017-019058 PMC5875648 29567843

[104] Cachemaille M, Grass F, Fournier N, Suter MR, Demartines N, Hu M, et al. Pain Intensity in the first 96 hours after abdominal surgery: a prospective cohort study. 2019;0(0):1–11. 10.1093/pm/pnz156 31322667

[105] Wood GJ, Shega JW, Lynch B, Von Roenn JH (2007). Management of intractable nausea and vomiting in patients at the end of life: “I was feeling nauseous all of the time … nothing was working”. JAMA.

[106] Büttner M, Walder B, von Elm E, Tramèr MR (2004). Is low-dose haloperidol a useful antiemetic?: A meta-analysis of published and unpublished randomized trials. Anesthesiology.

[107] Weschules DJ, Maxwell T, Reifsnyder J, Knowlton CH. Are newer, more expensive pharmacotherapy options associated with superior symptom control compared to less costly agents used in a collaborative practice setting? Am J Hosp Palliat Care. 2006 Mar-Apr;23. 10.1177/104990910602300211 16572752

[108] Currow DC, Quinn S, Agar M, Fazekas B, Hardy J, McCaffrey N, Eckermann S, Abernethy AP, Clark K (2015). Double-blind, placebo-controlled, randomized trial of octreotide in malignant bowel obstruction. J Pain Symptom Manag.

[109] Hosie A, Agar M, Lobb E, Davidson PM, Phillips J (2014). Palliative care nurses’ recognition and assessment of patients with delirium symptoms: a qualitative study using critical incident technique. Int J Nurs Stud.

[110] Downar J, Delaney JW, Hawryluck L, Kenny L (2016). Guidelines for the withdrawal of life-sustaining measures. Intensive Care Med.

[111] Hosker CM, Bennett MI (2016). Delirium and agitation at the end of life. BMJ.

[112] Leonard M, Raju B, Conroy M, Donnelly S, Trzepacz PT, Saunders J, Meagher D (2008). Reversibility of delirium in terminally ill patients and predictors of mortality. Palliat Med.

[113] Wee B, Hillier R. Interventions for noisy breathing in patients near to death. Cochrane Database Syst Rev. 2008;2008(1):CD005177. doi: 10.1002/14651858.CD005177.pub2. PMID: 18254072; PMCID: PMC6478131. 10.1002/14651858.CD005177.pub2 PMC6478131 18254072

[114] Campbell ML. Terminal dyspnea and respiratory distress. Crit Care Clin. 2004;20(3):403–17, viii-ix. doi: 10.1016/j.ccc.2004.03.015. PMID: 15183210. 10.1016/j.ccc.2004.03.015 15183210

[115] Jennings AL, Davies AN, Higgins JP, Gibbs JS, Broadley KE (2002). A systematic review of the use of opioids in the management of dyspnoea. Thorax.

